# Supplementary material for: Intensive systolic blood pressure treatment remodels brain perivascular spaces: A secondary analysis of the Systolic Pressure Intervention Trial (SPRINT)
Source: Neuroimage Clin. 2023 Sep 23;40:103513. doi: 10.1016/j.nicl.2023.103513 (PMC10540038; doi:10.1016/j.nicl.2023.103513)

**Supplemental Material:**

Intensive Systolic Blood Pressure Treatment Remodels Brain Perivascular Spaces: A Secondary Analysis of the Systolic Pressure Intervention Trial (SPRINT)

**Supplemental Methods:**

MRI Acquisition: MRIs were acquired across 7 sites. For each participant, baseline and follow-up MRI were acquired at the same site. Acquisition parameters were harmonized and scanner performance was monitored quarterly with a phantom acquisition with an approved phantom. All scanners showed stability of phantom measurements throughout the trial.

MRI models used in the study included 3T Philips Achieva 3.2 (at 3 sites), 3T Siemens Tim Trio VB17 (at 2 sites), 3T Siemens Verio VB17 (at 1 site), and 3T Siemens Skyra VD11B (at 1 site).

T1 sequences had repetition time (TR) = 1900ms, echo time (TE) = 2.89 ms, field of view (FOV) = 250mm, slice thickness = 1mm, 176 sagittal slices, and a native resolution of 1mm isotropic. T2 sequences had TR = 3200ms, TE = 409ms, FOV = 250mm, slice thickness = 1 mm, 176 sagittal slices, and native resolution of 1mm isotropic. FLAIR images had TR = 6000 ms, inversion time = 2200 ms, TE = 285 ms, FOV = 258mm, slice thickness = 1mm, 160 sagittal slices, and native resolution = 1mm isotropic.

**Supplemental Results:**

Determination of the Frangi Threshold

PVS visual ratings were performed for 120 randomly selected participants to determine the optimal Frangi threshold and repeated twice by the same rater separated by 2 months. Intraclass correlation coefficient for intra-rater PVS visual rating was 0.80. PVS volume fractions were associated with PVS visual ratings for the centrum semiovale and basal ganglia combined (R= 0.51) (Supplemental Figure 2). Quality review of scans and PVS segmentations was also repeated for this sample resulting in a Kappa of 1.0 for inclusion/exclusion based on quality of scan or segmentation.

**Supplemental Table 1.** Excluded Participants

|  | **Overall** | **Excluded Baseline** | **Excluded Longitudinal^a^** |
| --- | --- | --- | --- |
| **N** | 670 | 60 | 289 |
| **Intensive:Standard** | 355:315 | 32:28 | 148:141 |
| **% Female** | 40% (271) | 43% (26) | 44% (128) |
| **Age (years)** | 67 ± 8 | 66 ± 8 | 67 ± 9 |
| **Race or Ethnic Identity:** |  |  |  |
| **Black** | 33% (220) | 38% (23) | 36% (105) |
| **White** | 66% (439) | 58% (35) | 61% (177) |
| **Hispanic** | 5% (36) | 10% (6) | 7% (21) |
| **Other** | 2% (14) | 3% (2) | 2% (7) |
| **Baseline SBP (mm Hg)** | 138 ± 17 | 141 ± 20 | 140 ± 17 |
| **Framingham Score** | 17 ± 2 | 18 ± 2 | 17 ± 2 |
| **Baseline Imaging Measures** |  |  |  |
| **WMH Volume (cm3; IQR)** | 3.2 ± 4.6 | 2.4 ± 4.5 | 3.2 ± 4.5 |
| **Brain Parenchymal Fraction (%)** | 82 ± 4 | 82 ± 4 | 82 ± 4 |
| **n with useable baseline T2:** | 610 | 0 | 229 |
| **PVS Volume (cm3)** | 5.49 ± 1.70 |  | 5.29 ± 1.66 |
| **PVS Volume Fraction (%)** | 1.23 ± 0.32% |  | 1.20 ± 0.32 |

^a^Participants excluded from the longitudinal analysis included 208 without follow-up MRI, 55 excluded based on the follow-up MRI, and 26 excluded based on the baseline MRI.

SBP: systolic blood pressure. WMH: white matter hyperintensity. PVS: perivascular space

**Supplemental Table 2: Imaging Metrics**

|  | Baseline | Longitudinal Analysis | | | |
| --- | --- | --- | --- | --- | --- |
|  |  | Intensive Baseline | Intensive Follow-up | Standard Baseline | Standard Follow-up |
| N | 610 | 207 | 207 | 174 | 174 |
| TICV (cm^3^) | 1384 ± 148 | 1387 ± 144 | 1389 ± 143 | 1401 ± 146 | 1402 ± 147 |
| TBV (cm^3^) | 1135 ± 115 | 1140 ± 112 | 1112 ± 109 | 1153 ± 113 | 1128 ± 115 |
| BPF (%) | 82 ± 4 | 82 ± 3 | 80 ± 4 | 82 ± 4 | 81 ± 4 |
| WMH Volume ± IQR (cm^3^) | 3.23 ± 4.6 | 5.65 ± 7.8 | 6.8 ± 9.1 | 5.54 ± 6.76 | 7.87 ± 9.7 |
| logWMH ± IQR | 0.092 ± 0.117 | 0.13 ± 0.12 | 0.15 ± 0.14 | 0.13 ± 0.11 | 0.17 ± 0.14 |
| Tissue ROI Volume (cm^3^) | 443 ± 49 | 445 ± 47 | 441 ± 45 | 449 ± 49 | 446 ± 49 |
| PVS Total Volume (cm^3^) | 5.49 ± 1.70 | 5.45 ± 1.75 | 5.31 ± 1.68 | 5.52 ± 1.67 | 5.42 ± 1.76 |
| PVS Fraction (%) | 1.23 ± 0.32 | 1.23 ± 0.33 | 1.20 ± 0.33 | 1.23 ± 0.30 | 1.21 ± 0.33 |
| PVS Count (n) | 383 ± 102 | 388 ± 104 | 370 ± 96 | 392 ± 98 | 378 ± 101 |
| Mean PVS Volume (cm^3^) | 0.0138 ± 0.0019 | 0.014 ± 0.0021 | 0.0138 ± 0.0020 | 0.0139 ± 0.0019 | 0.0138 ± 0.0020 |

TICV: total intracranial volume. TBV: total brain volume. BPF: Brain Parenchymal Fraction. WMH: white matter hyperintensities. IQR: interquartile range. ROI: region of interest. PVS: perivascular space.

**Supplemental Figure 1**. **Inclusion/Exclusion Flowchart**


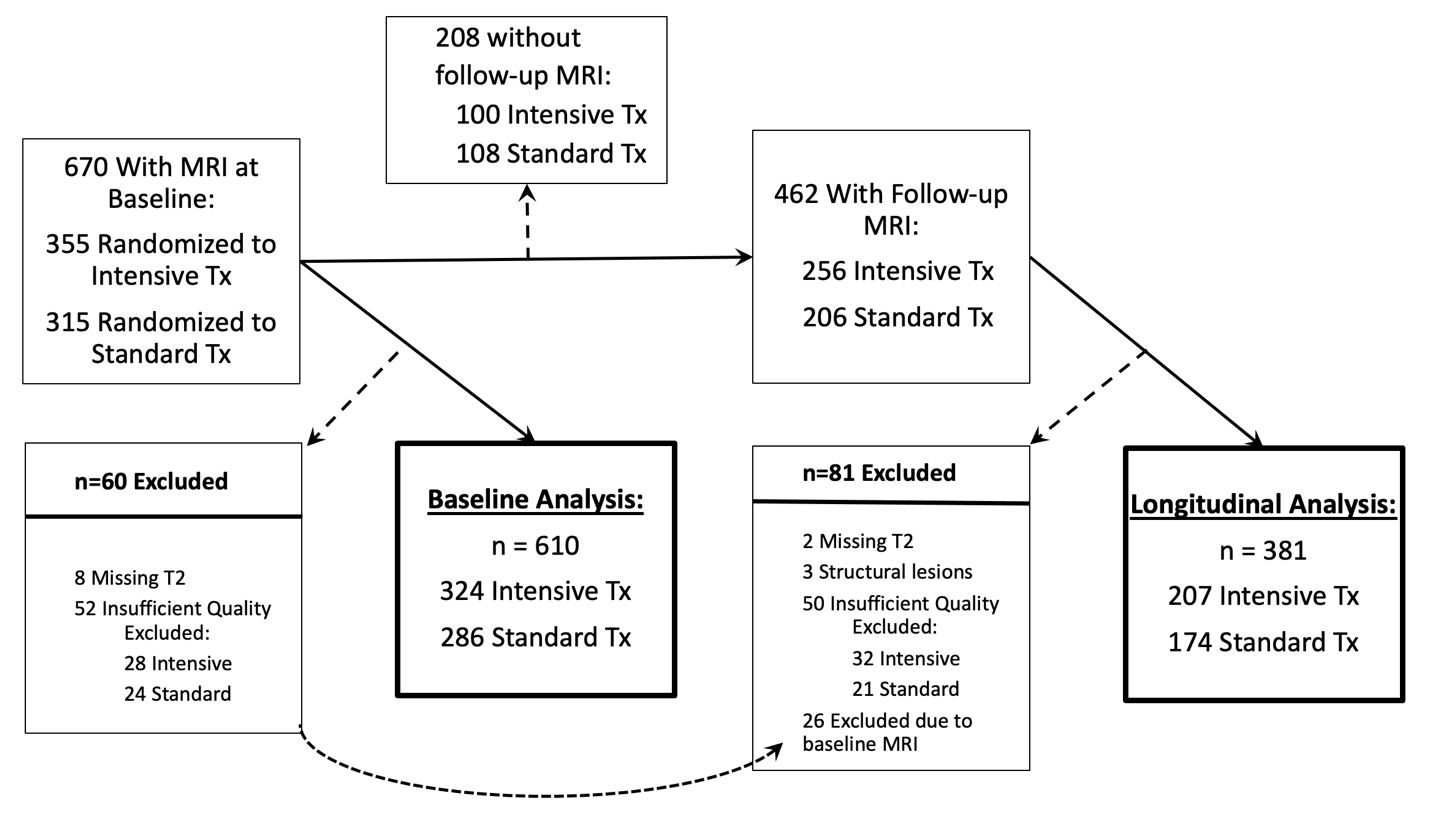


**Supplemental Figure 2.** **Determination of the Frangi Threshold**. A) For a subset of 120 randomly selected scans, the Frangi threshold was varied from 0.5 to 3.3, and at each threshold the Pearson’s correlation coefficient was calculated between the perivascular space (PVS) volume fraction and the PVS visual rating. The line plot is shown with the correlation coefficient on the Y-axis and the Frangi threshold on the X-axis. An optimal threshold of 2.7 was chosen after visual inspection and applied to the entire dataset. B) The association between PVS volume fraction and visual PVS ratings (combined centrum semiovale and basal ganglia) for 120 randomly selected scans, using the determined Frangi threshold of 2.7.


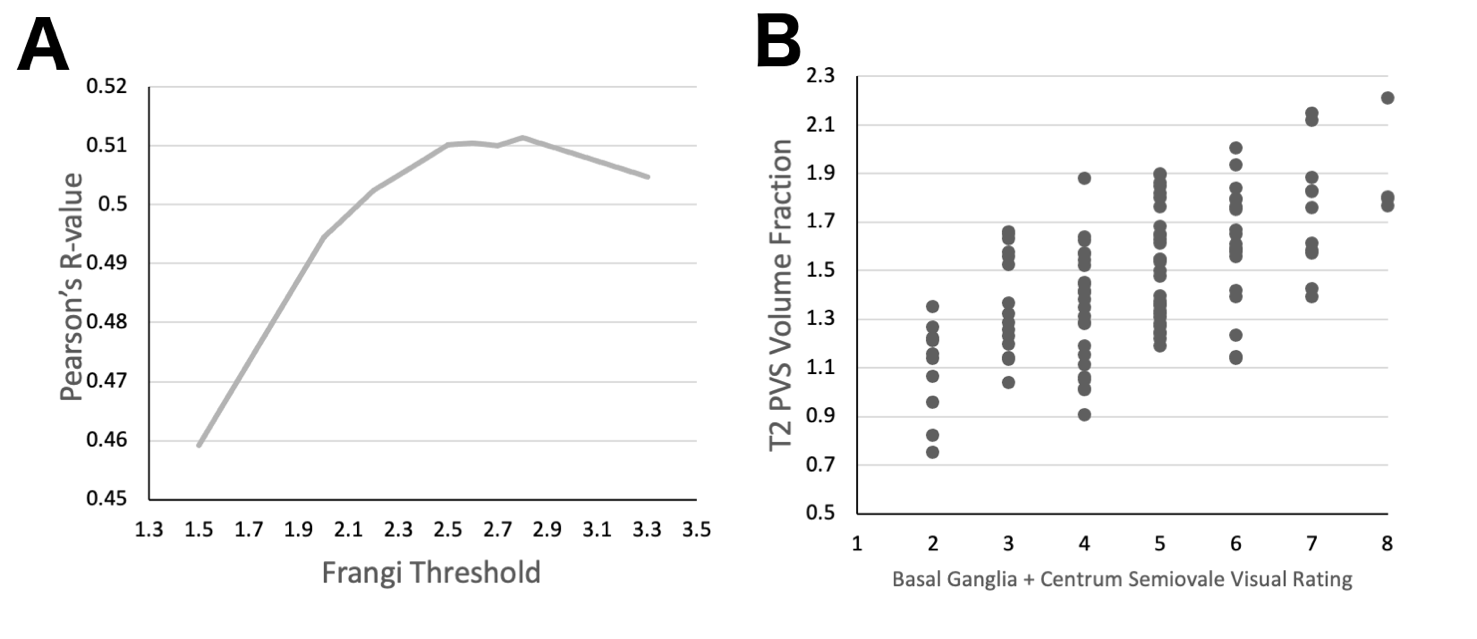


**Supplemental Figure 3**. **Achieved Systolic Blood Pressure (SBP) between treatment groups**. Achieved SBP is the area under the SBP curve over time, divided by the number of study days. The intensive SBP treatment group achieved a low SBP over the course of the study (p<0.001).


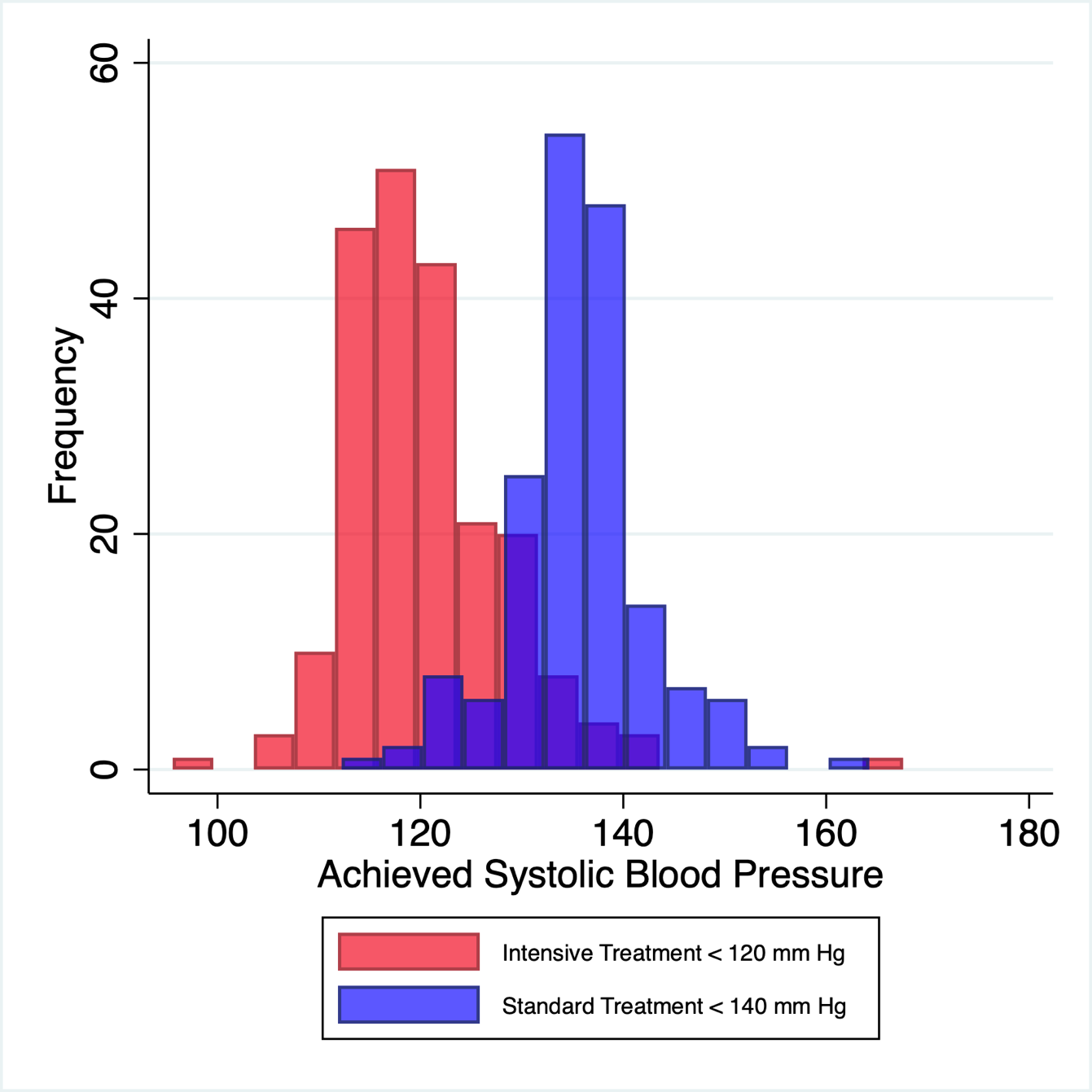

Supplement: Supplementary data 1 [file mmc1.docx]
